# Case of Pierson syndrome presented with hyphema,vitrous haemorrhage and subsequent neovascular glaucoma

**DOI:** 10.1186/s12886-023-02826-3

**Published:** 2023-02-24

**Authors:** Abdullah ALKhamees, Mansoor ALShemmari

**Affiliations:** AL-Bahar eye center, Ibn Sina Hospital, Kuwait city, Kuwait

**Keywords:** Pierson syndrome, Chronic kidney disease, LAMB2 mutations, Spontaneous Hyhpema

## Abstract

**Background:**

Pierson syndrome is a rare autosomal recessive disorder that causes congenital nephrotic syndrome, neurodevelopmental abnormalities, and several ocular signs. The Pierson syndrome is caused by a mutation of the LAMB2 gene, that encodes laminin beta 2, which is expressed in the glomerular basement membrane, in neuromuscular junctions, and within ocular structures. First described by Pierson et al., the ocular signs of Pierson syndrome include microcoria, which is most characteristic sign, as well as iris abnormalities, cataract, glaucoma, and retinal detachment.

**Case presentation:**

Herein, we report the case of a young female who, at 16 months, was diagnosed with congenital nephrotic syndrome, subsequently underwent a kidney transplant at age 4,did cataract surgery with IOL implantation in both eyes at age of 2 years and presented with ocular signs including high myopia, band keratopathy, t, nystagmus, retina, and optic nerve atrophy, she did not show nor did the family report any neurodevelopmental abnormalities. her genetic studies this missense variant c.970T< C p. (Cys324Arg) of LAMB2, later she developed spontaneous hyphema along with vitreous haemorrhage and increased intra ocular pressure in her left eye, she underwent cyclophotocouagulation to treat her high IOP.

**Conclusion:**

LAMB 2 mutations can be associated with multiple ocular signs that varies from mild to severe form, we are her to report our case who did not present with the typical ocular sign of microcoria for PS, did not have any neurodevelopmental  abnormality and presented with hyphaemia 2ndry to iris neovascularisation with vitreous haemorrhage with neovascular glaucoma.

## Background

Pierson syndrome is a rare autosomal recessive disorder that causes congenital nephrotic syndrome, neurodevelopmental abnormalities, and several ocular signs. The Pierson syndrome is caused by a mutation of the LAMB2 gene, located on chromosome 3 (3p21), that encodes laminin beta 2, which is expressed in the glomerular basement membrane, in neuromuscular junctions, and within ocular structures [1, 2]. First described by Pierson et al., the ocular signs of Pierson syndrome include microcoria, which is most characteristic sign, as well as iris abnormalities, cataract, glaucoma, and retinal detachment [3, 4].

Herein, we report the case of a young female who, at 16 months, was diagnosed with congenital nephrotic syndrome, subsequently underwent a kidney transplant at age 4, and presented with ocular signs including high myopia, nystagmus, and optic atrophy and later developed hyphema, vitreous heammorhage and neovascular glaucoma. DNA sequence analysis by Next generation sequencing analysis showed a homozygous missense variant c.970T < Cp. (Cys324Arg) in the LAMB2 gene that was previously reported by Falix et al. in 2017 [5].

## Case presentation

An 11-year-old girl presented to our paediatric ophthalmology clinic for an ophthalmic evaluation in November 2018. The patient had a history of nephrotic syndrome that was diagnosed at 16 months of age, had undergone kidney transplantation in other country at age 5 (no pathology study reports were available), and had been receiving dual immunosuppression with mycophenolate and tacrolimus (250 and 500 mg and 2 mg, in the morning and evening, respectively along with vitamin B complex and folic acid and thyroxin for her hypothyroidism). The patient had nystagmus since birth, bilateral high myopia (-7 -2 *180), and had undergone cataract extraction with posterior chamber IOL implantation at age 2 in our ophthalmology centre.

On examination, the bilateral ocular findings were as follows: vision 20/200 unaided, nystagmus, full ocular motility, posterior chamber IOL, band keratopathy, normal pupillary reflex on examination, tessellated fundus with macular hypoplasia and retinal thinning, optic nerve atrophy, and intraocular pressure of 18mmHg; cycloplegic refraction values were − 1.75*25 for the right eye and + 1.25*155 for the left eye.

**Fig. 1:**
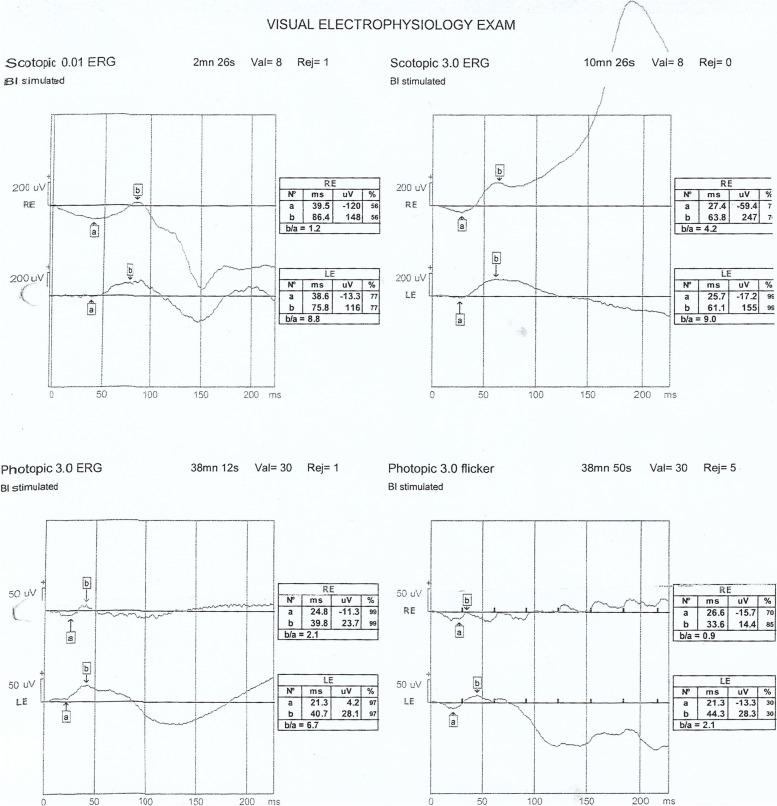
Her full-field ERG showed moderate attenuation of the amplitude and mild prolongation of implicit times of the rod-dependent responses as well as moderate attenuation of amplitudes and mild prolongation of implicit times of the cone-dependent responses in both eyes

**Fig. 2:**
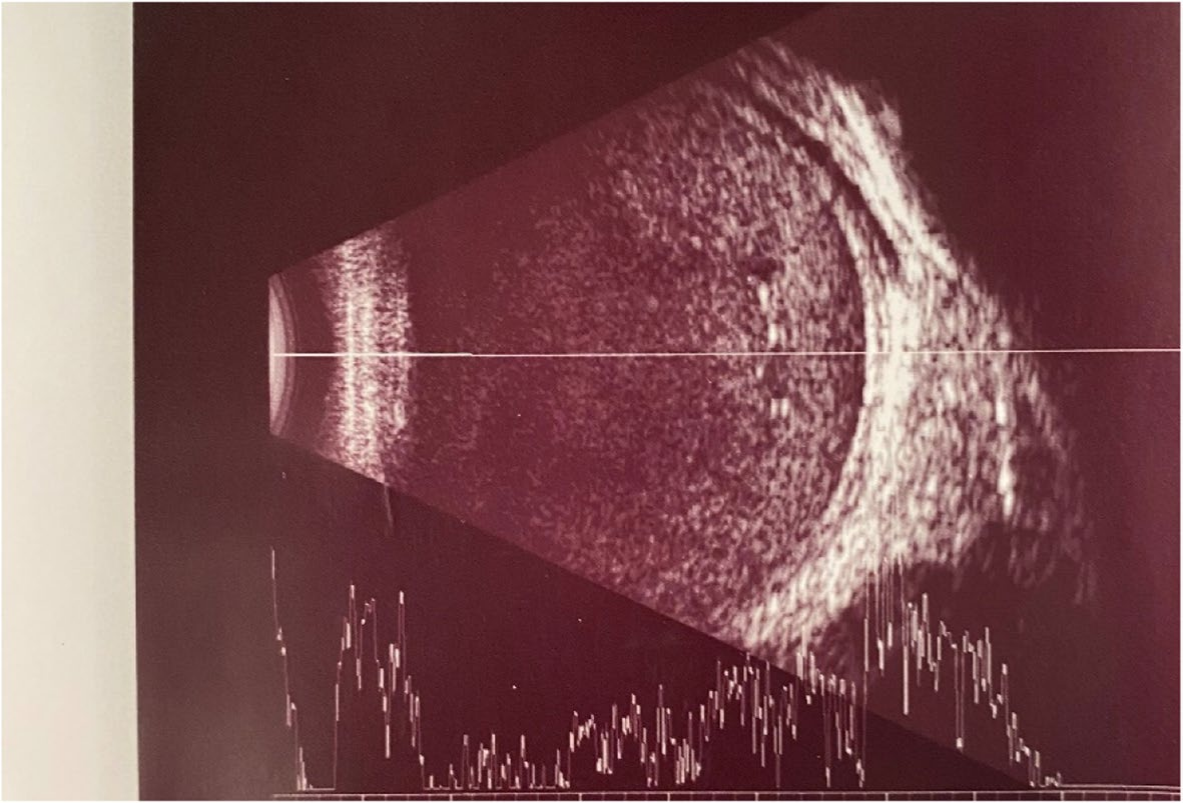
Ultrasonography of the left eye showing vitreous haemorrhage and no retinal detachment

**Fig. 3:**
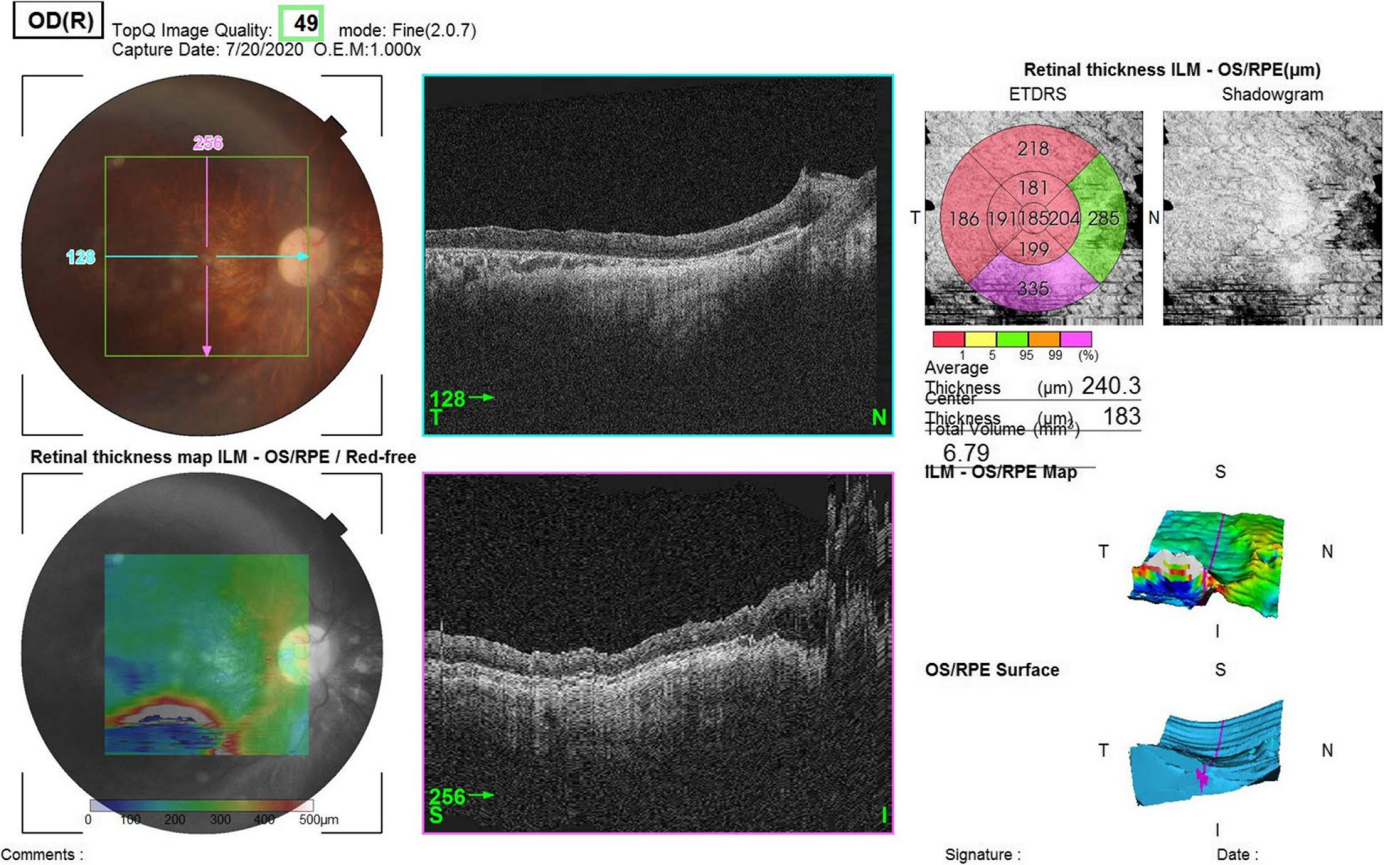
OCT of her right eye shows retina thinning, macular hypoplasia and undisguisable photoreceptors layer

**Fig. 4:**
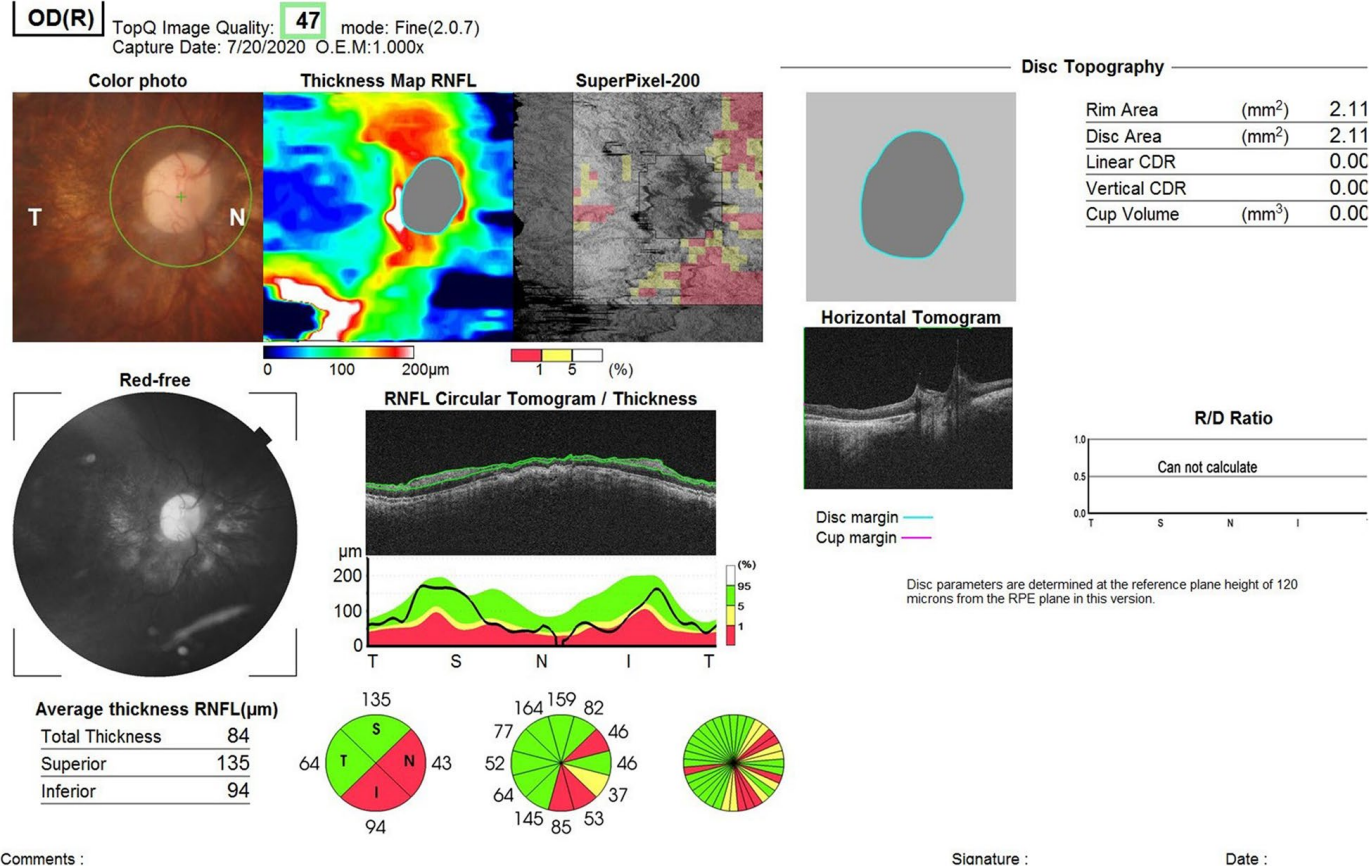
OCT of her right optic nerve shows optic nerve atrophy with abnormal vasculature emitting from disc

Accordingly, full-field ERG (Fig. 1) and genetic counselling were advised. The patient did not appear for a follow-up visit for 16 months but was referred from the emergency department with a complaint of pain in her left eye. Visual acuity examination showed no light perception in the left eye. Other findings for the left eye included spontaneous subtotal hyphaemia and intraocular pressure of 25mmHg, an ultrasound (Fig. 2) of the left eye was done and showed vitreous haemorrhage but without retinal detachment. Optical coherence tomography for the macula and the optic nerve was done for the other eye (Figs. 3 and 4). The patient had no history of trauma to the left eye, and was hospitalised and put on topical steroid drops, cycloplegics and aqueous suppressants. During her hospitalisation, a consultation to the retina service for the left vitreous haemorrhage; after careful discussion with the family and considering the visual prognosis, her family decided not to undertake surgical intervention due to her poor visual prognosis. However, despite treatment, her intraocular pressure increased to 55mmHg; an anterior chamber wash was performed, iris showed neovascularisation and the intraocular pressure decreased to the 24–26 mmhg range, but without any improvement in the visual acuity; though she experienced another episode of hyphema, the intraocular pressure remained stable.

Bloodwork-up coagulation profile of INR 1.06, APTT 29.4 s, and PT 15.7 s, tacrolimus level of 5.3 µg/L, Urea 4.6 mmol/L, Creatinine 114 mol/l, Alb 41 g/L, TSH7.1 mU/L, her Glomerular Filtration Rate 49 ml/min/1.73m2.

## Outcome and follow-up

In the left eye of the patient, the intraocular pressure continued to fluctuate and reached 55mmHg despite being on full glaucoma drops. In December 2020, she underwent cyclophotocoagulation for the left eye to reduce intraocular pressure and pain; follow-up in the next months showed an intraocular pressure of 25mmHg, with maintenance treatment with glaucoma drops, and no pain. Unfortunately, several months later, her left eye developed phthisis bulbi.

## Discussion and conclusions

The Pierson syndrome is a rare autosomal recessive disorder which causes congenital nephrotic syndrome, neurodevelopmental abnormalities and ocular manifestations that include microcoria (which our patient did not have), high myopia, nystagmus, iris hypoplasia and neovascularisation, ectropion uvea, cataract, persistent fetal vasculature, retinal findings include retinal ischemia, macular hypoplasia, neovascularisation retinal detachment, optic nerve atrophy, and glaucoma [4, 5]. The syndrome is caused by mutation in the LAMB2 gene located on chromosome 3 (3p21), that encodes laminin beta 2 which is expressed in glomerular basement membrane, neuromuscular junctions, and ocular structures [1, 2].

In this case, the patient had nystagmus and high myopia, and anterior segment examination showed bilateral band keratopathy, which is mostly due to her chronic renal disease, PC IOL, she didn’t have microcoria before cataract surgery, her fundus examination showed a tessellated fundus, OCT showed macular hypoplasia, retinal thinning, poor retinal lamination, undisguisable photoreceptors layer and optic nerve atrophy in right eye.

Our patient developed spontaneous hyphema with vitreous haemorrhage and increased intraocular pressure in her left eye, which we believe is due to ischemia with subsequent neovascular glaucoma, her right eye examination did not reveal any signs of neovascularisation. Her family members declined our recommendation for fundus fluorescein angiography to detect any ischemic changes in the right eye. patient was evaluated by retina specialist as we thought she may benefit from pars plana vitrectomy and laser treatment, but family also declined after discussion of visual prognosis and surgical complications. She did not show nor did the family report any neurodevelopmental abnormalities.

DNA analysis by next-generation sequencing followed by Sanger sequencing performed for the child and parents showed a homozygous missense c.970T < C p. (Cys324Arg) in axon 8 of LAMB2 gene in the affected child and a heterozygous missense variant of c.970T < C p. (Cys324Arg) of LAMB2 in both parents, who were apparently normal; this missense variant c.970T < C p. (Cys324Arg) of LAMB2 was previously reported (Falix et al.2017) This variant changed the highly conserved polar, non-charged Cysteine amino acid to a positively charged, basic polar hydrophilic arginine. The c.970T < C p. (Cys324Arg) mutation in the LAMB2 gene affects one of the eight highly conserved cysteine residues within the first EGF-like module of laminin b2 protein, these residues form disulfide bonds to achieve a correct 3D structure of the protein [5]. Falix et al. reported this mutation in a relatively mild variant of Pierson syndrome that was associated with later onset therapy resistant nephrotic syndrome. Hasselbacher et al. (2006) speculated that complete loss-of-function mutations in LAMB2 gene result in the Pierson syndrome, whereas missense LAMB2 mutations result in the congenital nephrotic syndrome, with or without ocular abnormalities [2].

In conclusion, we think that a better understanding of retinal association of Pierson syndrome may help in early diagnosis and anticipation of complications such as neovascular glaucoma after ocular surgery, ophthalmologist can offer intravitreal vascular endothelial growth factor inhibitor with cataract surgery and look for any signs ischemia and neovascularisation in the fundus or iris in subsequent visits witch can be managed with pan retinal photocoagulation, and offer glaucoma valve surgery for hight IOP or pars plana vitrectomy to manage retinal detachment.

## Data Availability

Data sharing is not applicable to this article as no were generated or during the current study.

## Abbreviations

LAMB β 2: laminin β 2
IOL: Intraocular lens
IOP: intra ocular pressure
PS: Pierson syndrome
Mmhg: millimetre of mercury
ERG: electroretinogram
OCT: optical coherence tomography
DNA: Deoxy ribonucleic acid
